# Multi-beam multi-slice X-ray ptychography

**DOI:** 10.1038/s41598-025-93757-0

**Published:** 2025-03-18

**Authors:** Mattias Åstrand, Ulrich Vogt, Runqing Yang, Pablo Villanueva Perez, Tang Li, Mikhail Lyubomirskiy, Maik Kahnt

**Affiliations:** 1 KTH Royal Institute of Technology, Department of Applied Physics, Bio-Opto-Nano Physics, Albanova University Center, 106 91 Stockholm, Sweden; 2 MAX IV Laboratory, Lund University, Box 118, 221 00 Lund, Sweden; 3 Department of Physics, Synchrotron Radiation Research, Lund University, Box 118, 221 00 Lund, Sweden; 4 Center for X-ray and Nano Science CXNS, Deutsches Elektronen-Synchrotron DESY, Notkestr. 85, 22607 Hamburg, Germany

**Keywords:** Ptychography, Multi-beam, Multi-slice, X-rays, Imaging techniques, Imaging techniques, Imaging and sensing, Phase-contrast microscopy, X-rays, Phase-contrast microscopy

## Abstract

X-ray ptychography provides the highest resolution non-destructive imaging at synchrotron radiation facilities, and the efficiency of this method is crucial for coping with limited experimental time. Recent advancements in multi-beam ptychography have enabled larger fields of view, but spatial resolution for large 3D samples remains constrained by their thickness, requiring consideration of multiple scattering events. Although this challenge has been addressed using multi-slicing in conventional ptychography, the integration of multi-slicing with multi-beam ptychography has not yet been explored. Here we present the first successful combination of these two methods, enabling high-resolution imaging of nanofeatures at depths comparable to the lateral dimensions that can be addressed by state-of-the-art multi-beam ptychography. Our approach is robust, reproducible across different beamlines, and ready for broader application. It marks a significant advancement in the field, establishing a new foundation for high-resolution 3D imaging of larger, thicker samples.

## Introduction

Advancements in numerous scientific fields, particularly in materials science and condensed matter physics, increasingly rely on innovations in microscopy. Detailed imaging of complex samples is vital for understanding their structural and chemical compositions as well as their functional properties. Although high spatial resolution can be achieved with scanning and transmission electron microscopy, these techniques are fundamentally limited to surface information, or to thin sample slices, owing to the shallow penetration depth of electrons. Consequently, non-destructive imaging of extended samples can only be achieved with hard X-rays, as these have a much higher penetration depth. Currently, the resolution of X-ray 3D microscopes is approaching that of transmission electron microscopes. In fact, thanks to the high spectral brightness of modern synchrotron radiation facilities and computational imaging methods such as ptychography^1,2^, it is now feasible to perform imaging with single-digit $\text{nm}$ resolution non-destructively^3^.

Conventional X-ray ptychography is a lens-less imaging technique. In fact, it does not require an objective lens to form an image, and it typically exploits focusing optics to increase photon fluence on the sample, thereby expediting data collection. The small yet intense X-ray beam that is formed by the focusing optics is an enabling factor for high quality imaging, as more photons given to the sample also mean more photons on the detector, a condition for higher resolution^4^. In essence, a sample is scanned through a focused beam, and diffraction patterns are collected on a far-field detector. That is, the distance of the detector from the sample means that what is measured is the intensity of the Fourier transform of the wavefront as it exits the sample. Using iterative algorithms, the complex-valued transmission function of the sample and the illumination function (probe) are reconstructed^1,2^. For ptychography to succeed, the beam must have a high degree of spatial and temporal coherence. Despite the high spectral brightness of fourth-generation synchrotron radiation facilities^5^, the spatial coherent fraction remains low at higher photon energies (it quickly drops to 10% at higher emittance)^6^, and a large portion of synchrotron radiation is wasted. This limitation has immediate implications for the duration of imaging experiments, the accessible field of view (FOV), or spatial resolution. Because many real-life, bulk samples are large, it is crucial to expand the standard X-ray ptychographic method to enable faster imaging of wider volumes in a non-destructive fashion, while retaining the high spatial resolution. Only in this way the imaging method can be used to its full potential of studying representative volumes in detail and aiding the characterization of extended, heterogeneous, and complex systems. We acknowledged that valuable research has been dedicated to develop different approaches to rapidly characterize large FOVs by means of ptychography, such as sparse scanning^7–9^ and fly-scanning^10–16^. Here we focus on the approach of using multiple X-ray beams in parallel, so-called multi-beam ptychography (MBP)^17–22^.

In recent years, it has been demonstrated that it is possible to use the otherwise unused incoherent fraction of synchrotron illumination. In MBP, multiple X-ray beams, coherent themselves but mutually incoherent, are implemented in parallel to scan over a sample. Consequently, more photons per diffraction pattern are recorded in the same amount of time, and larger areas of the sample can be covered than with single beam ptychography (SBP). This comes at the cost of increased computational effort, but disentangling diffraction data that originates from different beams is now a routinely solved problem^20–23^. The use of MBP thus enables larger FOVs to be imaged, meaning that larger samples are more accessible. This capability is exemplified by recent advances, which have successfully achieved the multi-beam imaging of an FOV exceeding ${100}{\ \upmu}\text{m}$^22^. However, because wide samples are often also thick, this recent achievement has raised the ultimate challenge for the characterization of larger 3D samples—overcoming limitations in resolution imposed by the imaging depth of field.

The more a sample extends in the direction of propagation of light, the higher the chance for multiple scattering interactions to occur while light is propagating through the sample. The assumption that multiple scattering events are negligible, which has been used to extend single-digit nm resolution tomography to samples beyond the depth of field of the imaging setup^3^, and to image large volumes with near-nanoscale resolution^16^, eventually becomes invalid. A different approach is necessary to enable high-resolution, thick-sample imaging, namely multi-slicing^14,24–34^. This is a method that accounts for the propagation of light through a sample and opens the possibility of resolving two or more so-called slices with data from a single scan. In practice, a thick sample in a multi-slicing scheme is regarded as a multitude of thin samples, separated by free-space propagation along the optical axis. Ultimately, the multi-slice model presents the possibility for enhanced lateral resolution when imaging thick samples. The additional information provided along the optical axis contributes to higher-quality volumetric reconstructions. Furthermore, multi-slicing can serve as a foundation for faster acquisition of tomographic datasets due to a reduced angle requirement^35–39^.

The path to high-quality, non-destructive imaging of extended samples is clear. Only hard X-rays offer highest resolution imaging when probing a bulk sample in depth. Multi-beam ptychography is a strong option for covering larger FOVs within reasonable experiment times, maximizing the use of the available synchrotron radiation. Multi-slicing is essential for managing the thickness of large samples. However, a critical obstacle persists: demonstrating the compatibility of multi-beam ptychography with multi-slicing. In this work, we address this challenge directly. For the first time, we demonstrate multi-beam multi-slice X-ray ptychography. We make use of an adaptive multi-beam X-ray ptychography setup^21^ and show that multiple samples, each consisting of two separate and distinctly different slices at a variety of separations along the beam axis, can all be resolved in their individual slices. This achievement paves the way for state-of-the-art non-destructive imaging of extended samples, offering resolution that is independent of sample thickness and a field of view that is unconstrained by sample width.

## Results

**Fig. 1:**
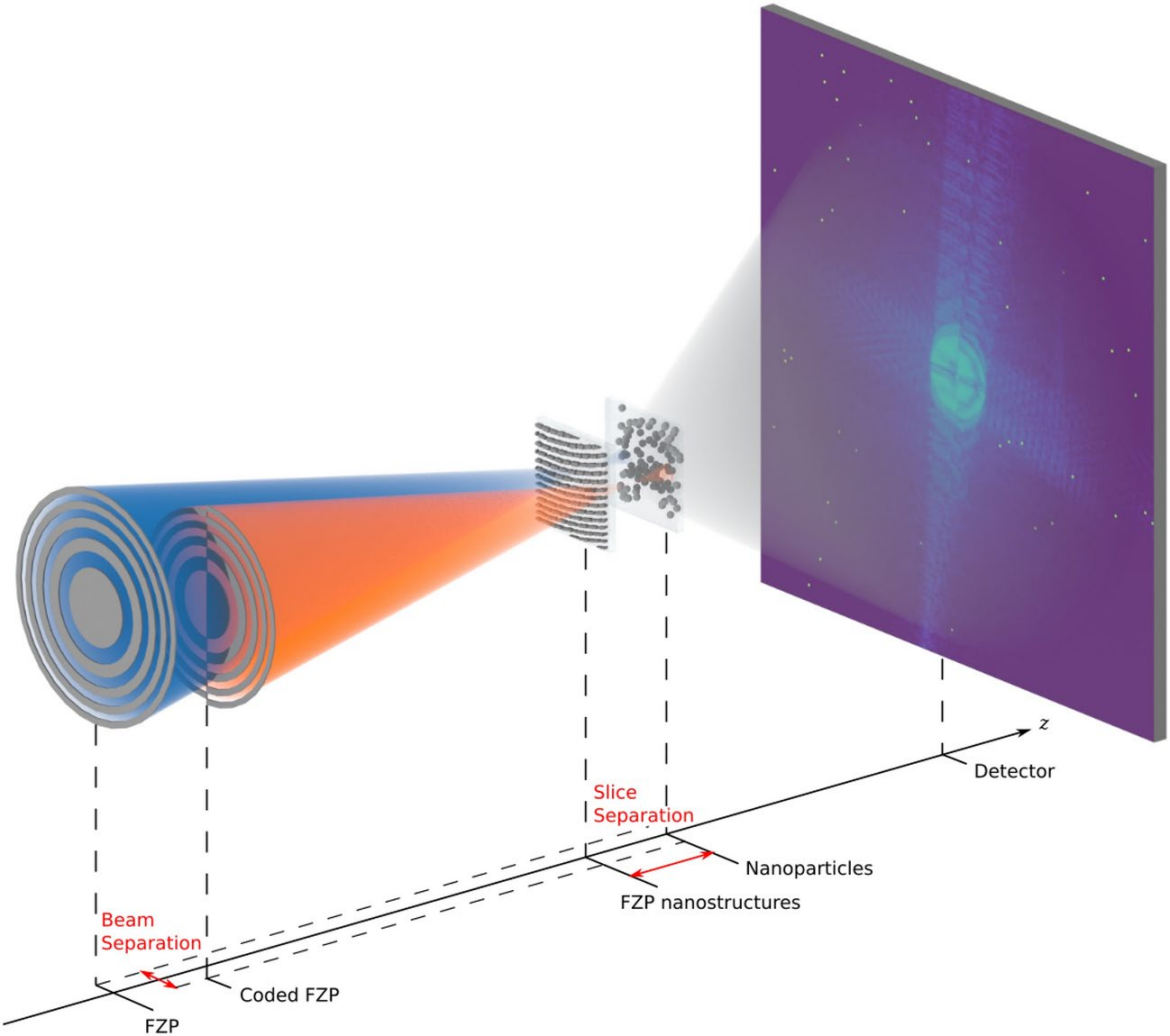
Schematic of the implemented setup with labels for the key parameters of a multi-beam multi-slice measurement. Two beams (blue and orange) are separated perpendicularly to the beam axis *z*. These illuminate nanostructures on two Si$_3$N$_4$ membranes that are separated in *z*. The upstream membrane features Fresnel zone plate nanostructures in gold, while the downstream membrane holds gold nanoparticles. The beam separation can be chosen at will, as the focusing optical elements (a standard and a coded Fresnel zone plate) are not laterally fixed. When the separation is set to zero, the beams overlap and act as one single beam.

In this study, we present the first demonstration of a multi-beam multi-slice ptychography experiment, conducted at the NanoMAX beamline (MAX IV Laboratory, Lund, Sweden)^40,41^. The experimental setup was analogous to that previously used for adaptive multi-beam X-ray ptychography^21^ and is schematized in Fig. 1. It consists of two Fresnel zone plates (FZPs) that are matched in numerical aperture, stacked and laterally separated to create two beams (also laterally separated) on the sample. To validate the reproducibility of our method and extend the initial findings, a second demonstration was performed at the P06 beamline of PETRA III (DESY, Hamburg, Germany)^42^. Measurements were carried out on samples comprising two distinct Si$_3$N$_4$ membranes, each featuring nanoscale structures as illustrated in Fig. 1. One membrane held gold nanobricks, at least 60 nm in width and up to 400 nm in height, arranged as the building blocks of an FZP. Although the FZP was not used for focusing, it served as a well-known reference sample. The second membrane was used to hold gold nanoparticles (NPs) of 200 nm in diameter.

**Fig. 2:**
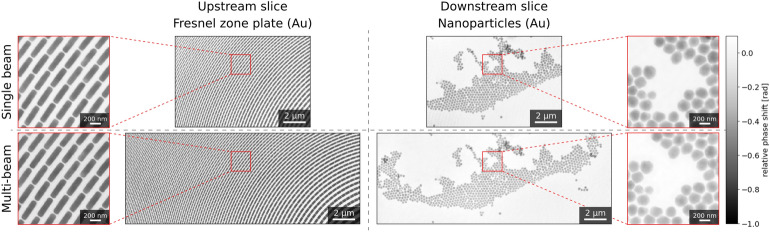
Multi-slicing ptychographic phase reconstructions for a two-layered sample with a slice separation of 700 $\upmu \text{m}$. SBP and MBP results are shown at the top and the bottom of the figure, respectively. To the left are reconstructions for the upstream slice, while the downstream slice is presented on the right side of the figure.

The experiment involved measuring samples at various separations between the Si$_3$N$_4$ membranes. Each measurement was carried out once using SBP, then again with MBP, as enabled by our adaptive setup^21^. The single beam scans served as a baseline for comparison with multi-beam scans. SBP and MBP measurements were taken over the same areas and with identical sampling parameters. Figure 2 provides a direct comparison of multi-slice reconstructions using SBP versus MBP on a sample with a 700$\ \upmu \text{m}$ separation between the Si$_3$N$_4$ membranes. The top panel of Fig. 2 shows the separated slices imaged with SBP, while the bottom panel shows the same slices imaged with MBP, demonstrating that MBP can successfully resolve the multi-slice problem, similar to SBP. A detailed comparison of ptychographic performance using a single or a multi-slice approach with both SBP and MBP is presented in the Supplementary information (see Fig. S1).

**Fig. 3:**
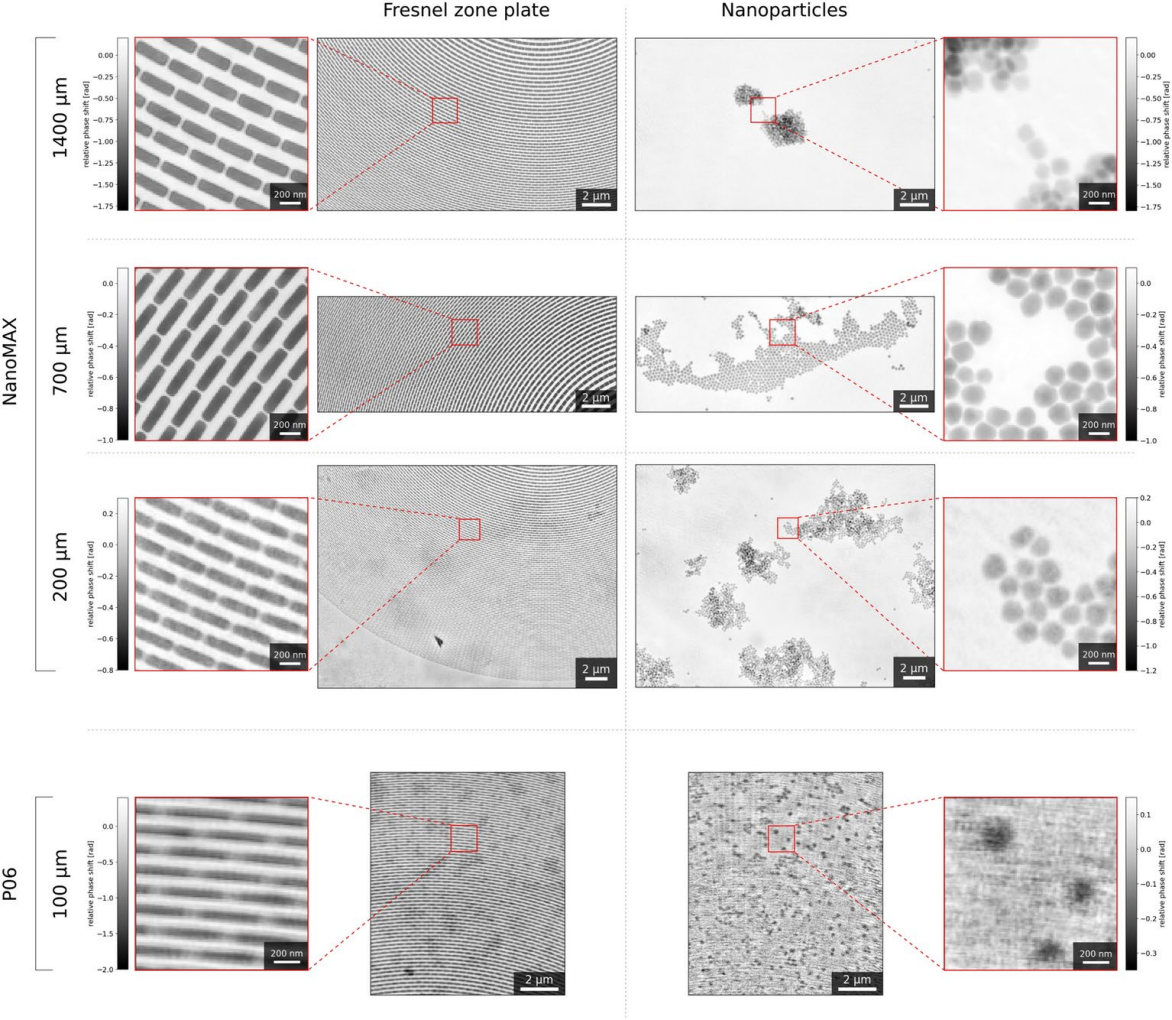
Multi-beam multi-slice ptychographic phase reconstructions for two-layered samples with various slice separations. The separations 1400 $\upmu \text{m}$, 700 $\upmu \text{m}$ and 200 $\upmu \text{m}$ were measured at NanoMAX. The smallest separation (100 $\upmu \text{m}$) was measured at P06. To the left are reconstructions for the slice with Fresnel zone plates, while the slice with nanoparticles is presented on the right.

To further demonstrate the applicability of multi-slicing within our MBP framework, we present phase reconstructions from a series of measurements on the same type of sample, but with varying slice separations. These results are shown in Fig. 3. In particular, the sample with the smallest spacing between the Si$_3$N$_4$ membranes was measured at the P06 beamline (DESY, Hamburg, Germany)^42^. The ability to resolve this small separation, relevant as it is comparable to the FOV approached in previous work on MBP^22^, at a different beamline underscores the versatility and robustness of our method.

The achieved resolution was estimated for all reconstructions and was in the range of 2 to 3 pixels regardless of the number of beams used in the ptychographic measurements. A more detailed discussion on resolution can be found in the Supplementary information (see Supplementary note 2). Furthermore, for all phase images, a quantification of the phase shift over corresponding sample features imaged by SBP and MBP, was carried out. Not only does the phase shift in single beam multi-slice correspond to that in multi-beam multi-slice ptychography, but the phase shift matches expectations (both theoretical and empirical), as discussed in the Supplementary information (see Supplementary note 3).

## Discussion

The sample dimensions considered in our experiments span several tens of $\upmu \text{m}$ and welcome the use of MBP for rapid imaging across larger FOVs. Features at varying depths within the samples require a multi-slicing approach to achieve high-resolution reconstructions. For the first time, we have addressed both of these challenges by combining MBP with multi-slicing. We successfully resolved all the slices in our various samples, as illustrated in Fig. 3, where reconstructions are shown for spacings as narrow as 100 $\upmu \text{m}$. Furthermore, based on analogous considerations with respect to resolution and thickness from the literature^28^, we extrapolate that it will be possible to resolve even thinner spacings, down to approximately 80 $\upmu \text{m}$ (see Supplementary note 2 in the Supplementary information for detailed calculations). If enhanced lateral resolution could be achieved, even thinner slice separations could be resolved, and this could help to address the current challenge of tomographic studies that are limited by the inability to treat samples as optically thin^3^. The extrapolated 80 $\upmu \text{m}$ limit, while hypothetical, should not overshadow the more practical achievement of successfully separating slices that are farther apart. Crucially, these slice separations provide a perfect match with the lateral sample sizes that can be handled using state-of-the-art MBP. The implications of our multi-slicing results for future MBP ptychotomography experiments are significant: the combination of MBP and multi-slicing will enable non-destructive, high-resolution 3D imaging of samples with diameters of 100 $\upmu \text{m}$ and beyond.

Although the contrast in reconstructions across smaller slice separations (200 $\upmu \text{m}$ sample from the NanoMAX series and 100 $\upmu \text{m}$ sample from P06) is sufficient for clear feature distinction, making high-resolution estimates possible, separating clean slices becomes increasingly challenging as the slice separation decreases. Specifically, we observe darker regions in the FZP slice that correspond to clusters of NPs in the other slice. This is because large features, such as clusters, correspond to low-frequency signals that can become confounded between slices when the propagation distance between them is short. Additionally, the reconstructions of the sample with the smallest slice separation (100 $\upmu \text{m}$) are generally less sharp than those of the NanoMAX series. This can be attributed to the more limited capabilities of the P06 beamline compared to NanoMAX for our experimental needs. The third-generation synchrotron radiation facility (PETRA III) housing the P06 beamline results in lower coherent flux of light in the probes, complicating the multi-modal decomposition in the iterative phase reconstruction process for P06 data analysis. Furthermore, the adaptive MBP setup was initially developed at NanoMAX, and the accumulated expertise provided a clear advantage for setting up and performing measurments at NanoMAX. Nevertheless, the resolution achieved for the P06 data is comparable to previous work on SBP multi-slicing at the same beamline^33^, confirming the capabilities of the experiment station.

As shown in Fig. 2, SBP and MBP phase reconstructions are comparable in both phase contrast and resolution. This indicates that our implementation of MBP on top of multi-slicing does not come at a cost in our current experimental procedure—there is sufficient sampling to separate information from distinct slices. Moreover, the successful demonstration of the compatibility of MBP with multi-slicing across different slice separations highlights the method’s robustness. For a more comprehensive comparison between SBP and MBP, we refer to the Supplementary information, which discusses SBP and MBP reconstructions for all samples and slices, as well as sample and slice locations along the beams. With these results in mind, and by accounting for different sampling conditions, this multi-beam multi-slicing success can be extended to separating more slices at reasonable separations in the future. That is, not only wider samples can be accessed by implementing more beams^22,23^, these can also be studied in more detail in depth. It follows that a path for future work exists and coincides with evaluating optimal experimental conditions for multi-beam multi-slice ptychography: understanding how many beams can be implemented, how different they should look, how many slices can be resolved, and what relation exists between these slices. A complimentary future approach to our work could be to use multi-slicing in combination with other approaches that enable the imaging of larger FOVs, with or without multiple X-ray beams—these are not mutually exclusive.

Overall, our experiment represents a pioneering demonstration of multi-beam multi-slice X-ray ptychography. This approach facilitates the study of thick and wide nanostructured samples without requiring modifications to existing MBP schemes. Software challenges associated with handling multi-beam multi-slice datasets had to be solved. A multi-slice routine was added to ptypy^43^ and is thus readily available to future users. Our demonstration is crucial to advance research beyond adaptive MBP. Recent efforts to standardize the use of multiple probes in MBP have enabled rapid scanning of FOVs on the order of 100 $\upmu \text{m}$^22^. This achievement, coupled with our multi-slice method’s applicability to samples of 100 $\upmu \text{m}$ in thickness and beyond, opens new possibilities for studying larger than ever tomographic samples while maintaining the highest spatial resolution.

## Methods

### Sample preparation

A series of multi-slice samples was fabricated, each consisting of two 200 nm thick Si$_3$N$_4$ membranes, which supported nanostructures positioned at different depths. In each pair of membranes that made up a two-slice sample, one membrane had Au FZPs, with zone heights of 400 nm and an outermost zone width of 60 nm^44,45^. The second membrane featured randomly adsorbed Au NPs with a diameter of 200 nm. To achieve the desired spacing between the Si$_3$N$_4$ membranes, Kapton foils of varying thickness (approximately 100 $\upmu \text{m}$) were used as spacers. Four multi-slice samples were prepared, with membrane separations of 100 $\upmu \text{m}$, 200 $\upmu \text{m}$, 700 $\upmu \text{m}$ and 1400 $\upmu \text{m}$. A visualization of such samples, as inserted and illuminated in our experimental setup, is given in Fig. 1.

### Microscope construction

Experiments were conducted at the NanoMAX beamline (MAX IV) and the P06 beamline of PETRA III (DESY). The experimental setup at both beamlines followed the principles of the design that was used in the demonstration of adaptive multi-beam X-ray ptychography^21^. Two numerical aperture-matched FZPs were stacked, each mounted on a separate motorized stage to allow independent movement. The upstream FZP had a diameter of 200 $\upmu \text{m}$ and the downstream FZP measured 196.4 $\upmu \text{m}$. Both were fabricated in gold, with a height of 400 nm and an outermost zone width of 60 $\text{nm}$. At an X-ray energy of 8 keV, the focal lengths of the upstream and downstream FZPs were 77.4 mm and 76 mm, respectively, accounting for the separation between them. The central axis of the lateral translation was defined by a tungsten wire, 25 $\upmu \text{m}$ in diameter, used as a central stop and positioned on the back of one FZP chip. A 15 $\upmu \text{m}$ slit cut in a 25 $\upmu \text{m}$ thick tungsten foil served as an order sorting aperture. Further downstream, the multi-slice samples were illuminated with probes of comparable sizes on both membranes. Illumination conditions are discussed in more detail in the Supplementary information (see Supplementary note 4 and Fig. S6). Diffraction data was collected using an Eiger2 X 4M detector^46^ (Dectris AG, Switzerland) at NanoMAX and an Eiger X 4M detector in vacuum^47^ (Dectris AG, Switzerland) at P06.

### Microscope operation

For single beam ptychography measurements, the FZPs were stacked conventionally. For multi-beam ptychography, the FZPs were laterally separated to form two distinct probes. At NanoMAX, the separation was horizontal and set to 8.575 $\upmu \text{m}$, while at P06, the separation was vertical and set to 6.746 $\upmu \text{m}$. These orientations and separations matched the orientation of the wire central stop and the alias cloaking conditions^20,21,48,49^ at each endstation, respectively. These configurations thus allowed for the collection of multi-slice data under both single and multi-beam ptychography conditions. The single beam ptychography results are consistent with earlier work on multi-slice X-ray ptychography at P06^33^, while the multi-beam ptychography results represent a novel advancement. Scanning was always done in steps, either with a Fermat spiral or a grid with randomly offset scanning positions. The dwell times per scan spot were of either 0.5 s or 2 s. Scanning parameters are collected and listed in the Supplementary information (see Table S1), while other details of the scanning procedures are available in our raw data files^50^.

### Phase retrieval

Diffraction patterns were cropped to 512 $\times$ 512 pixel, symmetrically around the center of the direct beam (pupil) on the detector. The cropped diffraction data was processed with the ptypy framework^43^, specifically, with 500 iterations of the ThreePIE engine^24^. This engine is a more generalized version of the ePIE engine^51^ and makes it possible to model samples as an ensemble of separate slices with free-space Fresnel propagation of the exit wavefront between the slices. Therefore, complex valued wavefronts of the multiple (two) probing beams, as well as the complex valued transmission function of each of the multiple (two) object slices, could be reconstructed for all samples in this work. Note that, starting from the first iteration of the algorithm, both object slices were updated concurrently. A collection of reconstruction parameters is found in the Supplementary information (see Table S2).

Reconstruction quality was refined through iterative adjustments of the geometric parameters of the experiment. Initially, the distance from the upstream slice to the detector was optimized for image sharpness in the upstream slice. Subsequently, the slice separation was varied to achieve optimal sharpness in the downstream slice. This led to a reconstructed slice separation that varied up to 30% from the nominal value (within reason when accounting for the glue used to assemble the samples). Once satisfactory reconstructions of both slices were obtained, the inital probe estimate was refined, followed by a re-evaluation of the upstream slice-detector distance and slice separation if necessary. Details of the MBP implementation can be found in previous work^21^.

## Supplementary Information

Supplementary Information.

## Data Availability

The raw dataset used for the results presented in this article is openly available at https://doi.org/10.5281/zenodo.13628121
