# Supplementary material for: Multi-beam multi-slice X-ray ptychography
Source: Sci Rep. 2025 Mar 18;15:9273. doi: 10.1038/s41598-025-93757-0 (PMC11920106; doi:10.1038/s41598-025-93757-0)
Supplement: Supplementary file 1 — Supplementary Information. [file 41598_2025_93757_MOESM1_ESM.pdf]

Multi-beam multi-slice X-ray ptychography

Supplementary information

Mattias Åstrand[1*], Ulrich Vogt[1], Runqing Yang[2,3], Pablo Villanueva Perez[3], Tang Li[4], Mikhail Lyubomirskiy[2], Maik Kahnt[2]

[1]KTH Royal Institute of Technology, Department of Applied Physics, Bio-Opto-Nano Physics, Albanova University Center, 106 91 Stockholm, Sweden.
[2]MAX IV Laboratory, Lund University, Box 118, 221 00 Lund, Sweden.
[3]Lund University, Department of Physics, Synchrotron Radiation Research, Box 118, 221 00 Lund, Sweden.
[4]Center for X-ray and Nano Science CXNS, Deutsches Elektronen-Synchrotron DESY, Notkestr. 85, 22607 Hamburg, Germany.

*Corresponding author(s). E-mail(s): maastra@kth.se;

Supplementary note 1 – Comparison with single slice

In Figure S1 we present all the possible combinations of ptychographic extensions that are presented in this manuscript. Specifically, we show the increase in the field of view (FOV) achieved by transitioning from single beam ptychography (SBP) to multi-beam ptychography (MBP). Additionally, we highlight the importance of multi-slicing for resolving features at different depths of our sample. When a single slice reconstruction is performed (as shown at the top of Figure S1), only one layer of the sample is resolved. In this case, the reconstruction predominantly captures the Fresnel zone plate (FZP) membrane of the sample only, while the presence of structures (nanoparticles) on the other membrane (at another depth) degrades the overall image quality. The slice with the stronger scattering structures (the FZPs in our samples) is the one most likely to be reconstructed when not attempting to separate layers within the sample. However, by initializing the probe estimate appropriately, recontructions can be made to focus on the nanoparticles, which scatter less, and have a blurred FZP in the background.

Note that single and multi-slice reconstructions following illumination with a single beam (left side in Figure S1) are reconstructions from the same dataset. Likewise, the single and multi-beam reconstructions for multi-beam illumination (right side in Figure S1) are also from a single dataset, but this dataset is different from the single beam one.

Supplementary note 2 – Resolution estimate

Resolution estimates were obtained by analyzing edge profiles and determining the width of the segment where the phase shift drops from 90% to 10%. This approach was applied to all phase reconstructions discussed in the main manuscript. Figure S2 outlines the step-by-step methodology used to estimate the resolution in both the single-beam ptychography (SBP) and multi-beam ptychography (MBP) reconstructions for the two slices of the 700 µm sample. The same process was used for other samples. Resolution estimates are plotted in Figure S3, along with the resolution limit (dashed black curve) suggested by Equation 1. This equation relates the sample thickness T (interpreted as the slice separation) to the X-ray wavelength λ and the resolution δr. This relationship has been discussed extensively in prior work [1].

$$T \leq 5.2 \cdot \frac{(\delta r)^2}{\lambda} \iff (\delta r)^2 \geq \frac{T \cdot \lambda}{5.2} \tag{1}$$

Using Equation 1, we can also extrapolate the smallest resolvable slice separation for a given lateral resolution in our multi-slice approach. Conservatively, using the largest value among the MBP resolution estimates in Figure S3, we calculate that slices as close as 84 µm can be distinguished.

Our findings indicate that SBP and MBP yield comparable results, with MBP being particularly attractive because of the substantial gain in FOV. Furthermore, the resolution achieved through multi-slice ptychography consistently outperforms the single slice limit, underscoring the necessity of multi-slicing for high-resolution ptychography in samples that exceed the depth of field of the imaging system. It is also evident from Figure S3 that the resolution over gold nanoparticles (NPs) is lower than over FZP features for both SBP and MBP measurements. This is expected, as FZPs are constructed with gold zones that have sharp, well-defined edges, while NPs are spherical, making it inherently more difficult to find sharp boundaries on them. Consequently, while the same fitting function is applied to both FZPs and NPs, the resulting profiles are sharper for FZPs and slightly more rounded for NPs, reflecting the inherent differences in their geometries.

Supplementary note 3 – Quantification of phase shift

The phase shift in the reconstructed images, following the illumination of samples with a single beam or two beams, is quantified. Specifically, phase shift profiles over corresponding features in images reconstructed from single beam and multi-beam data are compared in Figure S4. It is apparent that the profiles match well in the single beam and the multi-beam cases, with a phase shift that varies from 0.6 rad to 0.9 rad for FZP features, and from 0.3 rad to 0.4 rad for the NPs. The variance in the

FZP phase shift can be attributed to the nanofabrication (specifically, the electroplating) of the FZPs resulting in features of different heights in different samples. The variance in the NPs is small and the phase shift agrees well with the expected value from both theoretical calculations (suggesting 0.387 rad) [2]) and an isolated single slice measurement, performed with SBP on a part of a sample that consisted of NPs only (i.e., without X-ray scattering contributions from FZP features). The latter is presented in Figure S5, showing that a NP, not superimposed with a FZP feature, gives a phase shift of approximately 0.34 rad. The phase reconstruction in Figure S5 was obtained with a standard single beam, single slice approach. The reconstruction method implemented 2000 iterations of difference map algorithm, followed by 2000 iterations of maximum likelihood algorithm, analogous to previous studies [3].

Supplementary note 4 – Sample location and illumination

Measurements were carried out such that, based on the separation between the Si_3N_4 membranes, the size of the probing beam remained comparable on both membranes. This allowed for consistent sampling across the layers during scanning. Specifically, for the 1400 µm and 700 µm samples, the Si_3N_4 membranes were positioned symmetrically around the focal plane of the probing beams. In contrast, the 200 µm and 100 µm samples were placed with both membranes downstream of the focal plane. This strategy was adopted because positioning the membranes symmetrically around the focus in these thinner samples would result in a very small beam size, necessitating a large number of fine scanning steps. A visualization of these sample illumination conditions is presented in Figure S6.

Supplementary note 5 – Experimental parameters

The experimental parameters that are relevant to performing scans and reconstructions are presented in Table S1 and S2, respectively. These can also be found in the the raw data files analyzed in this study [4].

Expected SS [µm]	1400	700	200	100
Experiment site	NanoMAX	NanoMAX	NanoMAX	P06
Energy [keV]	8	8	8	8
Detector distance [m]	4.18	4.18	4.18	3.265
Detector pixel size [µm]	75	75	75	75
Scan pattern	Fermat spiral	Fermat spiral	Fermat spiral	Grid with randomized offsets (<0.5 jitter)
Scan size [µm^2]	12 × 12	12 × 8	18 × 8	5 × 5 (SBP), 10 × 5 (MBP)
Step size [nm]	500	300	900	500
Dwell time [s]	2	2	2	0.5
Number of diffraction patterns	670	1238	516	121 (SBP), 231 (MBP)

Table S1 List of scanning parameters that were used to measure the different samples.

Expected SS [µm]	1400	700	200	100
SS used in reconstructions [µm]	1580	700	250	100 (SBP), 130 (MBP)
Number of diffraction patterns	670	1238	516	121 (SBP), 231 (MBP)
Cropping	512 × 512	512 × 512	512 × 512	512 × 512
Engine	ThreePIE	ThreePIE	ThreePIE	ThreePIE
Number of iterations	500	500	500	500
Effective pixel size [nm]	16.87	16.87	16.87	13.17

Table S2 List of parameters that were used in the processes of image reconstruction.

References

[1] Tsai, E. H. R., Usov, I., Diaz, A., Menzel, A. & Guizar-Sicairos, M. X-ray ptychography with extended depth of field. *Opt. Express* **24**, 29089–29108 (2016). URL https://opg.optica.org/oe/abstract.cfm?URI=oe-24-25-29089.

[2] Gullikson, E. X-ray interactions with matter (1995). URL https://henke.lbl.gov/optical_constants/. Accessed: 2024-10.

[3] Åstrand, M., Kahnt, M., Johansson, U. & Vogt, U. Adaptive multi-beam x-ray ptychography. *Opt. Express* **32**, 22771–22780 (2024). URL https://opg.optica.org/oe/abstract.cfm?URI=oe-32-13-22771.

[4] Åstrand, M. *et al.* Raw data for ”Multi-beam multi-slice X-ray ptychography”. https://doi.org/10.5281/zenodo.13628121 (2024).

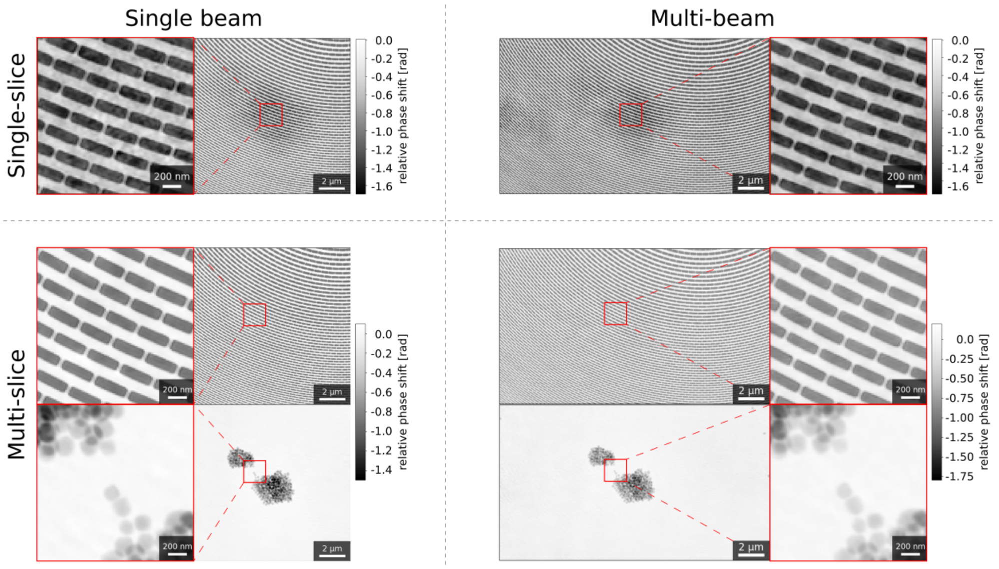

Figure S1 Reconstruction of the largest slice separation sample (1400 µm) with different ptychographic implementations. Specifically, we show single beam single slice (top-left), multi-beam single slice (top-right), single beam multi-slice (bottom-left), and the novelty of this work, multi-beam multi-slice ptychography (bottom-right).

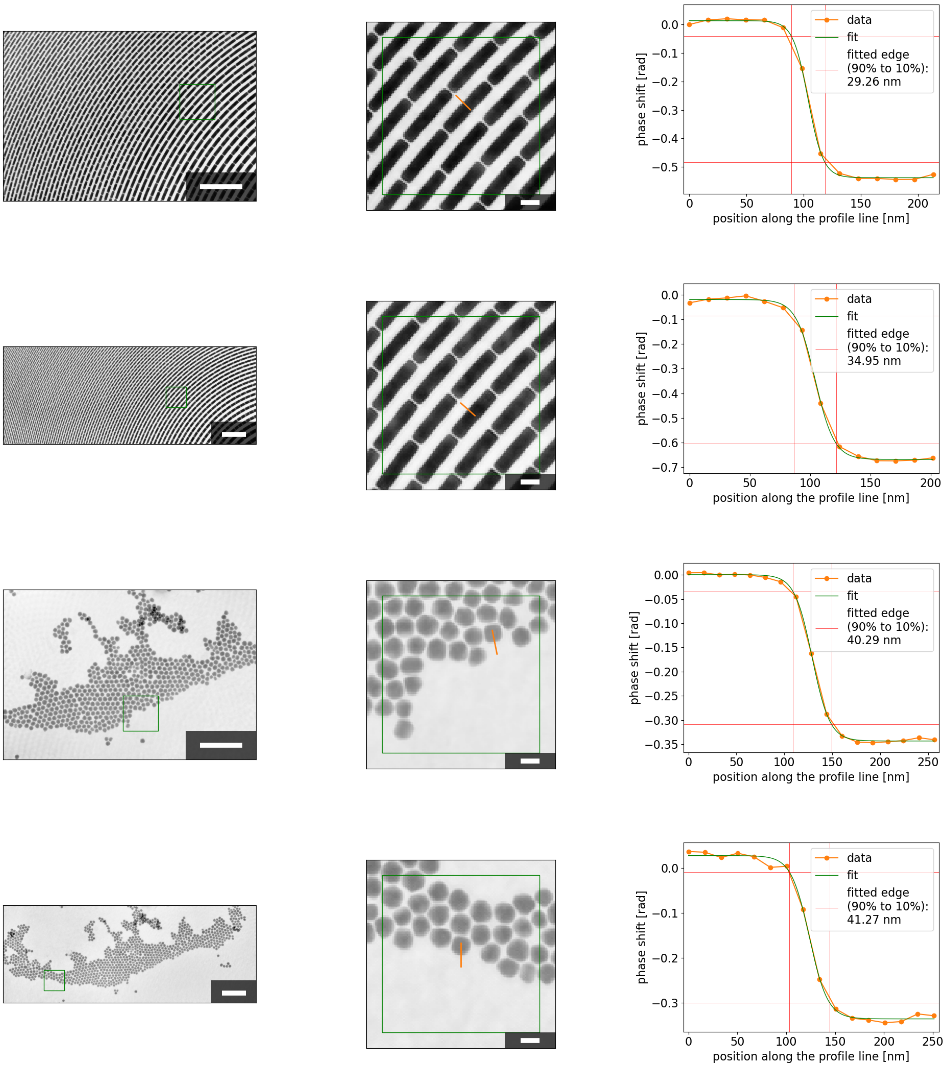

Figure S2 Analysis of sharp edges in the ptychographic reconstructions of the FZP and NP slices of the 700 µm sample for spatial resolution estimation. (left) An overview of the reconstructed slice. The scalebar indicates 2 µm. A sub-area is highlighted in green. (center) A zoom-in on this sub-area with a line (orange) that crosses the edge of a sharp feature. The scalebar indicates 200 nm. (right) The profile along this orange line is plotted and a fit is made to estimate resolution. Rows 1 and 3 are SBP results, while rows 2 and 4 are MBP results.

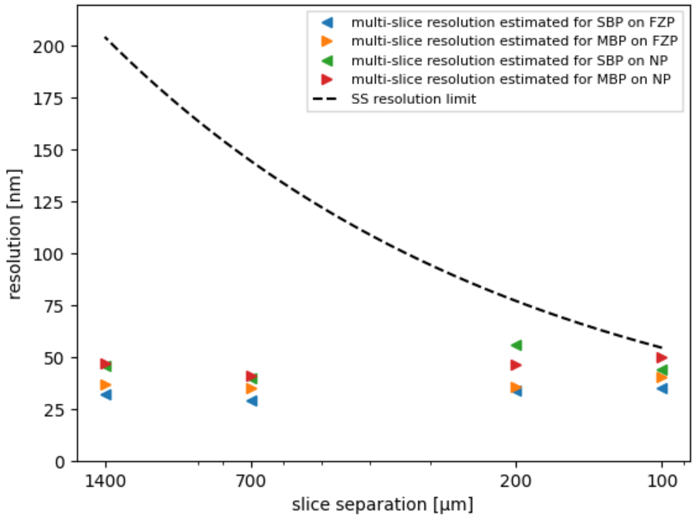

Figure S3 Estimates of resolution in the multi-slice reconstructions of this work. SBP and MBP estimates are calculated over sharp edges in the respective phase reconstructions, then color-coded and plotted next to each other. To provide a point of reference, the resolution limit for single slice reconstructions at 8 keV is calculated with Equation 1 and is also plotted (dashed black line).

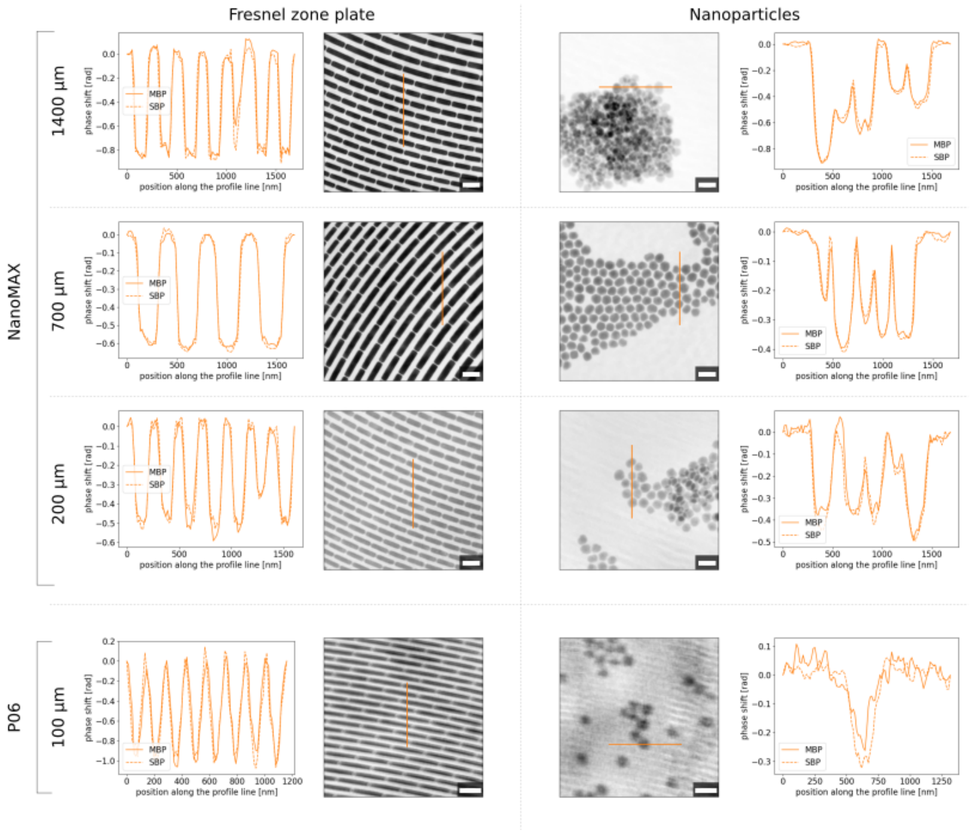

Figure S4 Reconstructed phase for the two slices in the different samples (the scalebars indicate 400 nm). In these phase contrast images, orange lines are used to indicate paths along which phase shift profiles are considered for both SBP and MBP. These profiles are plotted alongside the phase reconstructions.

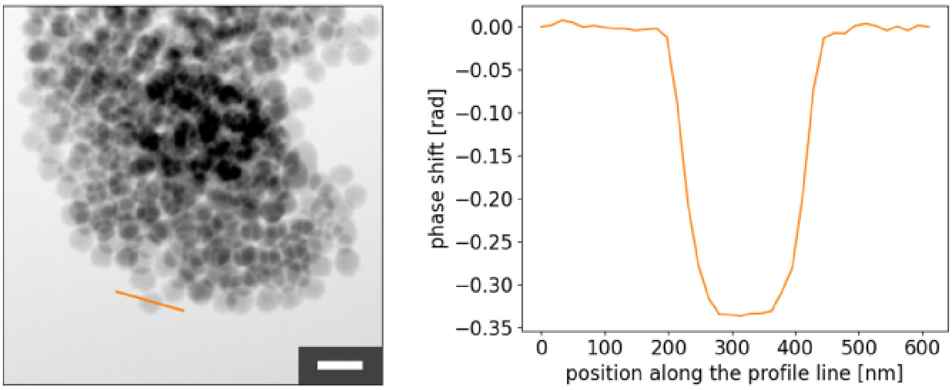

Figure S5 (left) Phase reconstruction of isolated NPs (not superimposed with FZPs) obtained with single beam single slice ptychography (the scalebar indicates 400 nm). This is the most classical approach to ptychography, known to be quantitative and thus a good basis for comparison with multi-beam multi-slice results. The phase shift over a single NP is quantified with a line profile, marked in orange in the phase reconstruction (left) and plotted (right). The phase shift value for a NP is estimated to 0.34 rad.

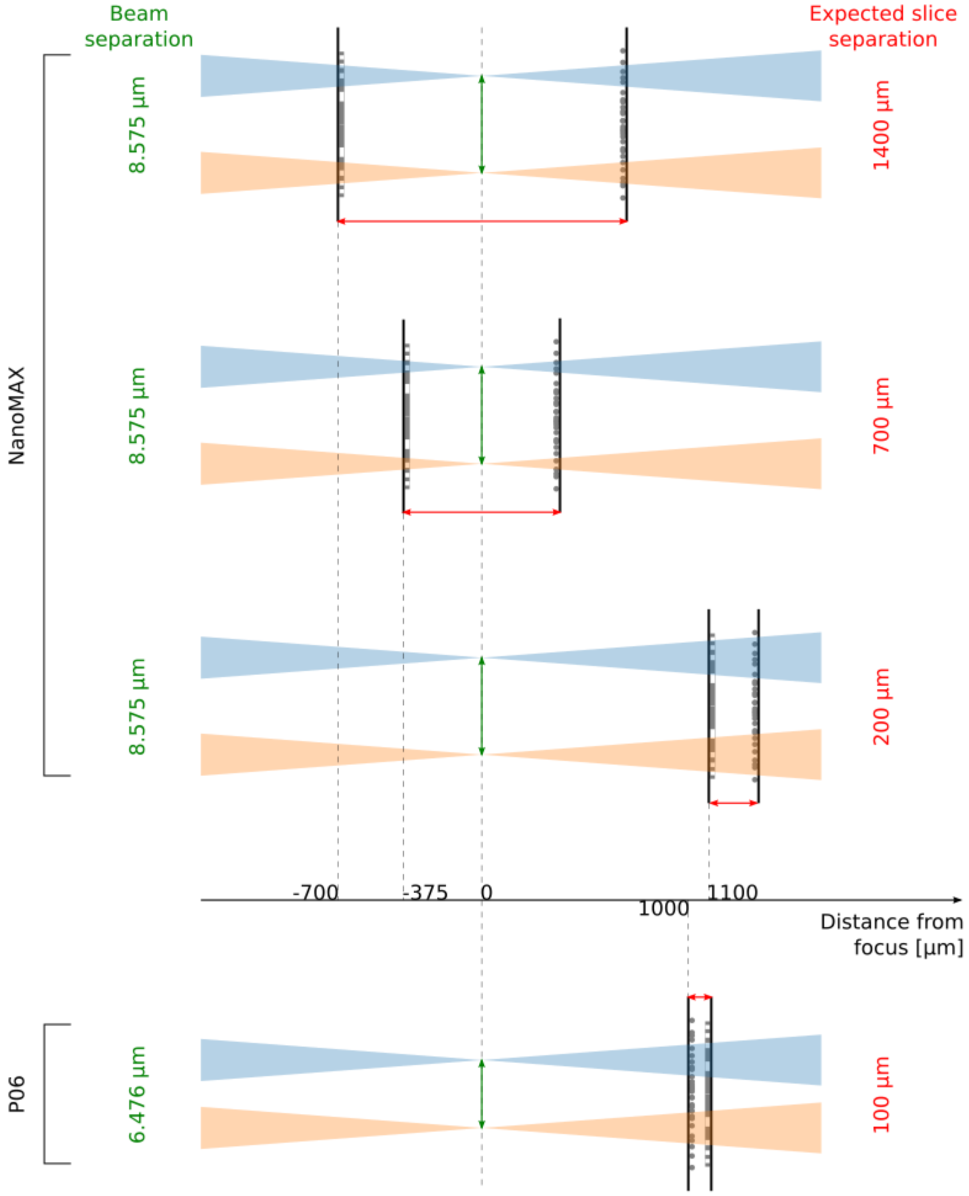

Figure S6 Visualization of the distinct measurements that were taken on the various samples presented in the main manuscript. As can be seen, the location of the samples relative to the focal plane of the beams varies as a function of membrane separation.
